# HOUSEHOLD COMPOSITION AND THE RESILIENCE OF OLDER ADULTS AGING IN COMMUNITY DURING COVID-19

**DOI:** 10.1093/geroni/igac059.1112

**Published:** 2022-12-20

**Authors:** Samara Scheckler, Jennifer Molinsky, Christopher Herbert

**Affiliations:** Harvard University, Cambridge, Massachusetts, United States; Harvard University, Cambridge, Massachusetts, United States; Harvard University, Cambridge, Massachusetts, United States

## Abstract

Household composition impacts older adults’ financial needs, earnings capacity, and benefits eligibility. It is also related to formal and informal LTSS access and may be associated with disparities between racial and ethnic groups since Black and Hispanic older adults are more likely than white older adults to live in multigenerational homes. The relationship between household composition and community-based aging is highly salient as multigenerational households and older people living alone are expected to increase in both number and share in coming years. This research framed the pandemic period as a stress-test to detect differences in resilience, defined as financial and LTSS stability and number of hardships, associated with older adults living alone, with partner, or with family or unrelated coresidents. Using the state-identified Health and Retirement Study (HRS), researchers developed a pre-pandemic profile of financial resources and public benefit utilization, informal and professional LTSS, and a vector of wellbeing measures by household composition type. Researchers then conducted two analyses to identify different pandemic experiences by composition type. First, wave over wave variation was regressed by household composition, noting any increased rates of instability in the 2020 wave. Researchers then used the HRS COVID-19 supplemental survey to describe pandemic hardships by household composition. About a third of HRS respondents lived in each household type. Findings, which offer a profile of resources by housing composition type and two analyses of resilience associated with composition, suggest complex relationships between household composition and resilience, but overall, residents living alone appear more vulnerable to instability.

